# I’ve got to be independent’: views of older people on recovery following road traffic injury in New South Wales, Australia

**DOI:** 10.1186/s12889-020-09391-0

**Published:** 2020-08-26

**Authors:** Katherine Brown, Ian D. Cameron, Lisa Keay, Ha Nguyen, Lisa Dillon, Jagnoor Jagnoor, Rebecca Ivers

**Affiliations:** 1 The George Institute for Global Health, UNSW Sydney, PO Box M201, Missenden Rd, Sydney, NSW 2050 Australia; 2 Faculty of Medicine and Health, the University of Sydney, Sydney, NSW Australia; 3 John Walsh Centre for Rehabilitation Research, Sydney Medical School Northern, the University of Sydney, Kolling Institute, Royal North Shore Hospital, St Leonards, NSW Australia; 4 School of Optometry and Vision Science, UNSW Sydney, Sydney, Australia; 5 School of Population Health, UNSW Sydney, Sydney, Australia

**Keywords:** Road traffic injury, Qualitative research, Content analysis, Facilitators, Barriers, Recovery, Older people, Functioning, Disability, International Classification of Functioning, Disability and Health (ICF)

## Abstract

**Background:**

Mild to moderate road traffic injury (RTI) in people of working age is associated with limited recovery. Less is known about RTI recovery in older age. This study explored the perspectives and factors associated with recovery and health-related quality of life following mild to moderate RTI in older age in New South Wales, Australia.

**Methods:**

A qualitative study using content analysis was undertaken. Participants aged 65 or more years were purposively selected from a larger inception cohort study of health outcomes following mild to moderate RTI conducted in New South Wales, Australia. Semi-structured interviews were undertaken at approximately 12 or 24 months post-injury. Content analysis was used to code and analyse the data, with methodological rigour obtained by double-coding and discussing findings to reach consensus. Results were reported using the consolidated criteria for reporting qualitative research (COREQ).

**Results:**

Nineteen participants were invited to participate in the study of which 12 completed interviews. Data saturation was reached at the twelfth interview. Recovery experiences were diverse. Five main themes were identified: recovery is regaining independence; injury and disability in older age; the burden of non-obvious disability; the importance of support; and positive personal approaches.

Key facilitators of recovery were: regaining independence; support from family and friends; and positive personal approaches. Key barriers were: threats to independence; passive coping behaviours; non-obvious disabilities (chronic pain, psychological impacts); and reluctance to raise ongoing issues with General Practitioners. Threats to independence, especially not driving and self-care, appeared to have a more profound effect on recovery than physical functioning.

**Conclusion:**

Older people view injury as a threat to independent functioning. This is somewhat different to what younger people report. Regaining independence is key to older people’s recovery and health-related quality of life following RTI, and should be a key consideration for health professionals, services and supports working with this unique cohort. Greater efforts to help older people regain their independence following RTI are needed and can be facilitated by health professionals and appropriate service provision.

**Trial registration:**

Australia New Zealand clinical trial registry identification number ACTRN12613000889752.

## Background

Worldwide, up to 50 million people sustain non-fatal road traffic injury (RTI) each year [1] resulting in enormous health, social and economic consequences for individuals and society [1–4].

It is well-established that older people do not recover as well as younger people from major, severe and catastrophic injuries [5, 6] yet the majority of RTI are mild to moderate and include injuries such as whiplash, rib, sternum or limb fractures, and mild traumatic brain injury (mTBI). In Australia, whilst approximately 7000 older people (≥ 65 years) are hospitalised each year following RTI, representing 12% of all RTI hospitalisations,[7] evidence suggests that an additional 50% of RTIs are treated in the Emergency Department (ED) and not admitted to hospital [8].

The world’s population is rapidly aging [9]. This demographic shift is especially pronounced in higher-income countries such as Australia, where life expectancies at birth have been increasing for the past 20 years [10] and are one of the highest in the world [11]. Currently 15% of the population are aged 65 years or over and this percentage is projected to steadily increase for at least the next 30 years, [1, 12] with the largest proportion of population growth seen in older driver age groups [11]. These changes will have important implications for Australia’s population health and wellbeing, and health and care systems [10].

Today’s older Australians are healthier compared to previous generations and have an increased number of years living free of disability and profound core activity limitation [10]. Older Australians are also key contributors to Australia’s social and economic well-being [9, 10, 12]. Many older Australians have not yet retired, are semi-retired or participate in unpaid work, especially as carers and volunteer roles. Many older Australians lead active, independent, fulfilling and social lives. RTI could lead to negative health, social and economic consequences for individuals, family and friends and wider society.

Injury in older age is a serious and growing problem. Older people are less resilient to physical injury compared to younger people due to biological changes associated with ageing [13–15]. An Australian study by Beck et al. demonstrated a 4.3% annual increase in major trauma in older people over the 10 years to 2016 in a cohort where the second most common mechanism of injury was RTI (22%) (second only to falls at 63%). Of particular concern, by 12-months 42% of the cohort had died and of the survivors only 28% were living independently [6]. Whilst a variety of injuries can result from falls, they are a low-energy trauma and common injuries such as hip fractures have a generally straightforward and predictable treatment, recovery and rehabilitation course. In contrast, RTI are a high-energy trauma that result in a diverse range of injuries. These include musculoskeletal injuries such as whiplash, lower back injury, fractures, mild traumatic brain injury and high rates of psychological distress, [16] including post-traumatic stress disorder (PTSD ) [17].

It is well established that older people do not recover as well compared to younger people from major, severe and catastrophic injuries due to any cause, including RTI [13, 14]. Evidence suggests older people have poorer long-term general health and functional outcomes 2 years after a mild to moderate RTI compared to younger adults [18]. Research from the United States on older people (≥ 65 years) treated in the ED following RTI found persistent pain was common and associated with functional decline, activity limitations and difficulties with activities of daily living, reduced self-rated health and changed living arrangements [19].

Pain itself presented a barrier to recovery, associated with increased bed rest or reduced activity 6 months after injury [20]. Psychological injuries were common and clinically significant post-traumatic stress disorder (PTSD) was present in 21% of older people at 6 months leading to increased risk of persistent pain, functional decline, and new disability [21].

More recently it has been hypothesised that the complex process of recovery from mild to moderate RTI is best understood from a holistic, biopsychosocial approach. For example, in 2017 Gopinath et al. demonstrated biopsychosocial factors such as general health, catastrophising, pain, social support and compensation factors were better prognostic indicators of recovery than injury type or location, [22] whilst in 2018, a systematic review by Samoborec et al. concluded multiple biopsychosocial factors influenced recovery. The strongest associations occurred between poor recovery and high initial pain intensity; pain duration, severity and catastrophising; and pre-injury physical and mental health [23]. A subsequent qualitative study by Samoborec et al. in 2019 was consistent with this evidence, finding recovery was multifaceted, complex, and influenced by comorbidities including chronic pain, depression and anxiety [24]. Major barriers to recovery were also identified. These were inability to self-care and/or complete domestic duties, and inability to participate in recreational activities leading to frustration, dissatisfaction and for some was a perceived cause of depression [24]. Despite these important findings in general adult road injury populations, evidence specifically for older people is lacking, despite known poorer outcomes in older people compared to younger people following mild to moderate RTI [18]. An Australian study by Gopinath et al. investigated health outcomes, including health-related qualify of life following mild to moderate RTI and found poorer physical functioning and general health in older (65 years and over) compared to younger (17–64 years) people 24 months post-injury but no difference in mental functioning [18].

New South Wales (NSW) is the most populous state in Australia with almost 8 million residents, [25] including over 1 million licence holders aged 65 years and older [11]. The number of older drivers’ licences in Australia has increased 44% over the past decade and is now exceeding population growth [7].

In NSW the Compulsory Third Party (CTP) insurance scheme, also known as Green Slip insurance, is a compulsory requirement of motor vehicle registration. CTP insurance is purchased by the vehicle owner to insure themselves from their own liability for injuries or death of other road users caused by the fault of that vehicle. The scheme provides benefits for pedestrians, passengers, cyclists, motorcyclists, drivers of other vehicles and, to a limited extent, drivers at fault, injured in a motor vehicle accident in NSW [26]. This study uses a population-based cohort of older people who are NSW residents injured in an RTI. It includes people who lodged a claim for benefits, as well as those who did not claim or were not eligible for the scheme (such as those injured whilst on private land).

This study aimed to explore in-depth perspectives, and factors related to recovery and health-related quality of life following a mild to moderate RTI in older age. There is a need for exploratory research using qualitative methods to capture important aspects of recovery and health not captured by quantitative methods, [27] such as the previously unexplored influence of contextual factors on recovery and health following RTI in older age.

## Method

### Participants and setting

This study was a sub-study of the larger Factors Influencing Social and Health Outcomes following road transport injury quantitative inception cohort study (the FISH study) [28]. The FISH study cohort consists of people (aged 17+ years) who sustain a physical road traffic injury (RTI) (car occupant, motorcyclist, pedestrian, and bicyclist) in the state of New South Wales (NSW), Australia who have been recruited within one month of injury [28]. The study excludes severe and catastrophic injuries (such as severe traumatic brain injury, spinal cord injury, amputations and severe burns). The complete FISH study inclusion and exclusion criteria and recruitment details are described in Jagnoor et al. [28].

Purposive quota sampling of sex, age, and living arrangements was used to ensure maximal diversity of participants and ensure equal representation of participants in key characteristics [29]. Recruitment staff were provided with a sampling reference grid (Additional file 1) to assist with the quota sampling process. Participants were eligible for inclusion in the qualitative sub-study if they were currently enrolled in the FISH study, were aged 65+ years, and had completed either their 12-month or 24-month follow-up interview. An opt-in approach was used, whereby at the conclusion of the FISH study follow-up interview, participants were invited to participate in the sub-study. Participants who indicated they would like to participate were contacted by the interviewer to confirm they wished to participate and arrange for a study information sheet and consent form to be sent out. Participants were then contacted to arrange an interview time. Verbal informed consent was obtained prior to each interview by the interviewer reading out the consent form to the participant, and the participant stating whether they consented to participate in the study. Prior to commencing the study, ethical approval to conduct the study, including the procedure for obtaining verbal informed consent, was obtained from the University of Sydney Human Research Ethics Committee. Co-authors with extensive experience in qualitative research in injury estimated data saturation, that is, the point at which no new categories or concepts could be found when analyzing the interview transcripts, [30] would occur between 10 and 15 interviews.

### Design

The study was conducted using directed qualitative content analysis methodology, using the approach outlined by Hsieh and Shannon. This approach consists of three main steps: coding the data into categories, identifying themes and patterns, and transforming the findings into a summary of key results [31]. Content analysis categories were informed by the conceptual model of the International Classification of Functioning, Disability and Health (ICF). The ICF model is widely used in qualitative studies related to injury, functioning, disability and older age, including brain injury, [32] spinal cord injury, [33] acquired contractures in older people, [34] and home rehabilitation in older people [35]. In the RTI literature, Samoborec et al. explored barriers to recovery using the biopsychosocial model from which the ICF was developed [24].

The ICF is based upon the biopsychosocial model of disability, in which functioning and disability are outcomes resulting from dynamic interactions between health conditions, concepts describing functioning and contextual factors [36, 37]. The ICF defines functioning as it is influenced by health conditions. Health conditions can directly or indirectly influence body structure and function, activities and participation [37]. Functioning is described in terms of body structures and functions (physical, psychological and cognitive functions, and pain) as well as activities and participation (including self-care, mobility, daily activities and social participation). Contextual factors are divided into environmental factors (physical, social and attitudinal factors, including support, systems, services and policies) and personal factors (including personal attributes, life experience and general health) [36].

### Data collection

In-depth semi-structured phone interviews were conducted. An interview guide (Additional file 2) was developed and used to facilitate exploration of all aspects of the ICF. Interviews ranged in between 10 and 50 min duration and continued until data saturation was reached at the twelfth interview, confirmed by no categories or concepts emergent from analysis of interview transcripts [30]. The primary author transcribed the first interview and the remaining interviews were transcribed by a professional transcription service. All interview transcripts were quality checked by listening to and reading the transcriptions word by word. Any discrepancies were scrutinised. The de-identified data were stored on a password-protected secure network drive at The George Institute for Global Health, Sydney.

### Data analysis

Transcripts were read line-by-line to become familiar with the data. Information relevant to the aim of the study was then identified and categorised into the ICF categories described above. Given the complex, multi-dimensional nature of recovery, some data was applicable to more than one theme and appropriate illustrative quotes were chosen. Preliminary themes and patterns occurring within and across categories were identified from the transcripts by the primary author by reading and interpreting the data. Methodological rigour and coding consistency were ensured by having a second author cross-check category assignment, preliminary and final themes. Discrepancies were discussed and a consensus reached. Data analysis was an iterative process with ongoing collaboration with co-authors. All authors reviewed and approved the results.

### Reporting results

The study was carried out in accordance with the consolidated criteria for reporting qualitative research (COREQ) 32-item checklist [38]. Study findings were reported in the following sections:

i) Participant characteristics (Table 1)
ii) Overview of recovery experiences
iii) Perspectives and themes, reported by ICF concept [31] (Tables 2, 3, 4, 5 and 6)
iv) Summary of key findings (Table 7).

**Table 1:** Participant characteristics (*n* = 12)

		***N***
**Sex**	Male	5
	Female	7
**Age (at injury)**	65–69 years	4
	70–74 years	5
	75–79 years	1
	80–84 years	1
	85+ years	1
**Marital status**	Married / de facto	7
	Divorced / widowed / separated	4
	Never married	1
**Education**	Secondary	5
	Technical / Other	1
	Tertiary / University	6
**Claimed compensation**	Yes	5
	No	7
**Living alone**	Yes	4
	No	7
	Unknown	1
**Role in crash**	Driver	6
	Passenger	2
	Pedestrian	1
	Cyclist	1
	Motorcyclist	2
**Hospitalisation**	Yes (> 12 h)	7
	Emergency Department only	3
	No / not known	2

**Table 2:** Illustrative quotes for Theme 1 - Recovery is regaining independence

*P11 (85–89 years, upper limb dislocation)* *‘I had of course to feed myself with my left hand, do everything with my left hand as I had no capacity in my right hand. But look, I got through that … really it was just a matter of letting it heal … I suppose it was worse for my wife who had to do the driving and do the shopping and things like that’.*	
*P10 (65–69 years, multiple arm fractures)* *‘I couldn’t live on my own because I couldn’t do anything for myself... I couldn’t cut my food, I couldn’t drive … I couldn’t do anything, so I had to go and live with [my daughter] permanently which wasn’t my choice’.*	
*P1 (70–74 years, whiplash)* *‘I don’t have enough strength in my arm to be able to start the lawnmower, so unless someone comes and starts it for me the lawn doesn’t get mowed, you know?’*	
*P5 (70–74 years, leg injuries)* *‘I got up and had my shower each day, very slow, I could hardly walk … I said [to the nurses], “No, I want be independent, I’ve got to use my legs”’.*	
*P5 (70–74 years, leg injuries)* *‘I walked around the shops today... [for] maybe an hour and half … I mean the walking’s not helping but I think it is helping somewhere inside because it is exercise every day. You need to be able to walk and do those things.*	
*P3 (70–74 years, head injury)* *‘I still couldn’t drive for about three weeks. They just wanted to make sure that everything was okay … in case there was a recurrence or something, which is fair enough. But it annoyed me because I wanted to drive’.*	
*P4 (70–74 years, fractured sternum)* *‘I felt hesitant the first time because where I lived, I always have to go through this roundabout. So, the very first time, yes, I was a bit hesitant, but I thought, no, I’ve got to do it. So, I’m just probably a little bit more careful or cautious could I say. But after that I was fine’.*	
*P2 (80–85 years, upper & lower limb injuries)* *‘I hadn’t normally until very recently needed help. I was showering, dressing and that sort of thing. But since … the pain and problems have come back … the last fortnight I actually do need a bit of help dressing. Now that’s never happened before in my life. When you’ve been just picking up things for 85 years, you know, suddenly to say, “Now don’t pick that up, or don’t reach for that” it’s very, very difficult’.*

**Table 3:** Illustrative quotes for Theme 2 – Injury and disability in older age

*P10 (65–69 years, multiple arm fractures)* *‘So, you can’t hit my arm and it’s really painful … I couldn’t drive for six months … I couldn’t lift the grandchildren … that was a huge problem and it still extremely hurts when I lift them on my arm’.*	
*P2 (80–85 years, arm / leg injuries)* *‘I was already suffering from a neuropathy … and also Parkinson’s and so this has really exacerbated it, compounded it … I’m typing [on the computer] instead of writing … fortunately the brain is still reasonably accessible’.*	
*P1 (70–74 years, whiplash)* *‘It’s getting harder to do [social activities and sport] because, I mean I do catch up with them, like for a barbecue and things like that, but it’s not the same sort of situation where we used to go out and we – we play a round of golf and have two beers and come home and things like that’.*	
*P11 (85–89 years, upper limb dislocation)* *‘Really, I mean, I’m now doing everything … I’m not terribly good on managing a crowbar these days and digging a deep hole, but otherwise I’m doing everything’.*	
*P6 (70–74 years, fractured ribs)* *‘I do have pain, but you know, I am at an age now, that you can’t do without any pain, but I would say it’s got nothing to do with that [the injury]’.*	
*P10 (65–69 years, multiple arm fractures)* *‘I am not quite sure what retirement means****.*** *I tend to do more than I ever did but I have retired … [the injury] accelerated it. Yeah, I wouldn’t have [retired] because I was actually working with my daughter and minding the children and doing other things and that stopped me from doing that’.*	
*P12 (65–69 years, fractured sternum, whiplash, psychological impact)* *‘I had a mortgage and I’m on my own, so I had to go back earlier... It just got to the point where I felt totally burnt out’.*	
*P12 (65–69 years, fractured sternum, whiplash, psychological impact)* *‘When I went back to work after my accident, the fear was, oh my God, I have to pay this mortgage off, and I’m going to pay it, it’s not much, but I had to pay it off, and I did’.*	
*P8 (75–79 years, head injury, arm movement limitation)* *‘When I’m doing something, I can remember what I’m doing, but given half an hour, nowadays, I’ve forgotten it... that’s why I thought I had Alzheimers and I wanted the test’.*

**Table 4:** Illustrative quotes for Theme 3 – The burden of non-obvious disability

*P1 (70–74 years, whiplash)* *‘I also don’t drive a car anymore. I’m just paranoid about driving a car and I won’t sit in the back of a car’.*	
*P5 (70–74 years, leg injuries)* *‘I was scared when my husband was driving. I don’t know whether it was me or - I don’t know … I was quite scared’.*	
*P7 (65–69 years, mild traumatic brain injury)* *‘I really don’t like thinking about it, you know. It’s had a psychological impact … quite probably a significant psychological impact’.*	
*P8 (75–79 years, head injury, arm movement limitation)* *‘Oh, one of the things that’s really important and I don’t know why or anything but since the injury my right shoulder, I can’t lift my arms very well. Yeah, that didn’t appear to be injured in the accident’.*	
*P12 (65–69 years, fractured sternum, whiplash, psychological impact)* *‘When I came home, I had a bit of stiffness in my neck … I didn’t really worry about it too much... I thought no, my neck will settle down. But I found over the last two years it’s [my neck] gradually getting worse’.*

**Table 5:** Illustrative quotes for Theme 4 – The importance of support

*P10 (65–69 years, multiple arm fractures)* *‘It’s good to have support, that’s the main thing. I feel sorry for people that don’t have support … I have a daughter who was wonderful … so, I had that support with her, and I had some nice friends around that gave me support. That helped a lot’.*	
*P2 (80–85 years, upper & lower limb injuries)* *‘It has been a big change [for my wife]. Obviously, it’s been a worry... she does drive me around more than she used to... [and] she’s been helping me with getting my shirt on and everything’*	
*P4 (70–74 years, fractured sternum)* *‘I suffer from benign vertigo and I’d been bending over, packing a lot of boxes and I kept having minor attacks of it … I had a friend; they would stand me up beside a chair with a box on it and they’d put everything on the table so I wouldn’t have to bend over and I wouldn’t have to lift … it was really lovely’.*	
*P2 (80–85 years, upper & lower limb injuries)* *‘I’ve found people very, very helpful actually... on one occasion someone came up to me and said, “I’ve seen you standing there for a while. Do you need any help?”’*	
*P7 (65–69, mild traumatic brain injury)* *‘[Psychologically] I think there’s stuff lingering there. Yeah, I think there’s an aftermath. [I’d prefer to] just not think about it. There’s nothing he [GP] could do. [Laughs] there’s nothing he would do’.* *P8 (75–79 years, mild traumatic brain injury, arm movement limitation)* *‘[my GP is] a good doctor but he doesn’t seem to think that women are very useful [laughs]’* *P5 (70–74 years, leg injuries)* *‘[I injured] my legs which [the doctors] never, ever did a thing for in hospital. All they were worrying about was the other injuries that weren’t visible … they said, “Don’t worry about [your legs], that’s your last problem”. I thought it is not the last problem, if there’s nothing wrong with my heart, I need my legs … I think if I’d had treatment on my legs earlier, I wouldn’t be in this pain and suffering now’.*	
*P1 (70–74 years, whiplash)* *‘just after the accident I had quite a few falls... I went to Stepping On and did that program and I’ve only had one fall since then’.*	
*P2 (80–85 years, arm / leg injuries):* *‘it did affect my attitude crossing the road, and particularly in crowds …*. *the insurance company has paid for some counselling … … so I’m not too bad there.’*	
*P1 (70–74 years, whiplash)* *‘It was just an annoying pain continuously … … the insurance company agreed to physio, and then they cut the physio out and I’ve been in pain ever since … my solicitor said everything should be straight forward, that they were making a claim and I should get money to go and continue with physio’.*	
*P1 (70–74 years, whiplash)* *‘[One thing that I will say, I’m very annoyed with the other driver’s insurance company]...[they] sent me to see another orthopaedic surgeon and he said there was problems on … not the left hand side but the right hand side, which was totally not right … and now … I got a letter … … I’ve got to see another orthopaedic surgeon. And then a psychologist’.*	
*P2 (80–85 years, arm / leg injuries)* *‘When I’m out socially I find I’m using taxis quite a lot. Which is a bit of an expense. So, anything to do with the accident I can claim back. But going off to do a bit of ordinary shopping I can’t obviously’.*	
*P1 (70–74 years, whiplash)* *‘It’s all public transport or my wife will drive me or my step-daughter or my daughter will come and pick me up... if they are not available, I just get public transport. It’s only about a six minute walk to the railway station. And there’s plenty of buses around’.*	
*P2 (80–85 years, upper & lower limb injuries)* *‘Where we live it’s quite well served by buses … if it stops at the normal stops it’s not a big problem. But the other day... I ended up being hauled into the bus by the bus driver and pushed into the bus by a passer-by... yes, it’s not too good for morale that. But it does work’.*

**Table 6:** Illustrative quotes for Theme 5 – Positive personal approaches

*P7 (65–69, mild traumatic brain injury)* *‘They said [in the Emergency Department] the problem is I am too stoic. So that can really be against you [laughs]. It really can. I often resent wearing [helmets] but I feel like framing that one [laughs]’.*	
*P9 (65–69 years, chest injuries)* *‘I know they [ribs] are there but I wouldn’t class it as pain … in the end you just do things that you know you can do. I can’t work as hard as I could, but I do what I have to do... I just discourage people giving me big hugs’.*	
*P2 (80–85 years, upper & lower limb injuries)* *‘At the time I thought things weren’t too bad. And it’s interesting to me that I totally underestimated how much this had affected me. On the other hand, having laid in hospital beds for a while and looked at other people, I’m not too bad’.*	
*P9 (65–69 years, chest injuries)* *‘I don’t employ anyone. There were lots of jobs I could do even straight away … at certain times, different times [my injuries] affect me a fair bit but basically, I’m back to full work’.*	
*P9 (65–69 years, chest injuries)* *So-called pain killers, I just went off them as quick as I could a long way short of what some people would because I just got the shits with [the] up and down feeling … [you don’t need] pain killers you just do things that doesn’t hurt … I’ve never been a great believer in pain killers.*	
*P10 (65–69 years, multiple arm fractures)* *‘For the first time I was just going to make sure I had my own place and yeah so pretty exciting. I got all new furniture and everything and I never lived there … I am back now and have family all around me now … so it worked well, I suppose’.*	
*P10 (Female, 65–69 years, multiple arm fractures)* *‘It is just sort of like watching and being aware. You are always aware … so it’s always on your mind but it won’t stop running my life. [We] have to live, don’t we? At least I didn’t have any other serious injuries. I just have lots of scars up my arm, that’s all’.*	
*P10 (65–69 years, multiple arm fractures)* *‘I mean I do a lot of walking, but you are always very cautious of not tripping. So, it hasn’t stopped me from doing anything that I want to do. Not anymore’.*

**Table 7:**
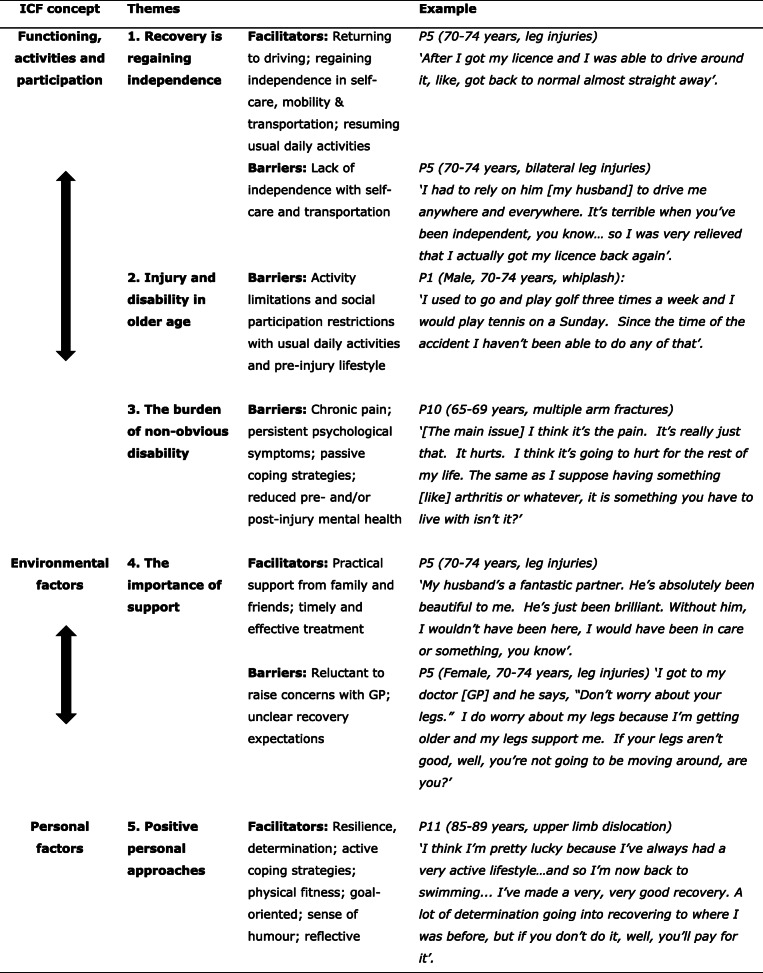
Themes, facilitators and barriers of RTI recovery in older age, based on ICF concepts.

## Results

### Participant characteristics

A total of 19 eligible participants were invited to participate in the study. Of these, 12 people completed an interview and 7 people did not (4 participants were unable to be contacted; 2 declined to participate; and 1 had difficulty hearing over the phone) (Table 1). Data saturation was reached by the 12th interview, confirmed by no new categories or concepts emergent from the analysis of interview transcripts. Participants had sustained a variety of injuries, including limb fractures, whiplash, rib fractures, sternum fractures or mild traumatic brain injury (mTBI). Psychological impacts such as anxiety, depression and post-traumatic stress disorder (PTSD) were reported.

### Overview of recovery experiences

Recovery experiences, trajectories and outcomes were diverse, reflecting the different types of injuries in the study cohort; pre-injury health status, and individual lifestyles and priorities.

Some participants fully recovered from their injuries:

*P4 (Female, 70–74 years, fractured sternum).*

*‘I’ve always felt pretty good … [so]... once I got over the cracked sternum, my life carried on like usual’.*

However, for other participants, their injury and recovery experience were life-changing, and resulted in major disruptions to their lives:

*P10 (Female, 65–69 years, multiple arm fractures).*

*‘Well, it is all very traumatic having had many surgeries, which was terrifying. I would be in hospital, a long way away from the family … that was a real big problem … I lost my car; it was written off. So, the day that I had the accident I was going to my new unit I had just rented... so I paid rent for six months on a house I never lived in... so, it was all pretty crappy’.*

The degree of disability reported by participants varied. One participant described major limitation in activities following bilateral soft tissue leg injuries, despite this being a ‘minor’ severity injury:

*P5 (Female, 70–74 years, leg injuries).*

*‘I didn’t do hardly any chores or anything in the house because I couldn’t move properly. I had to learn to walk again. It took me all my time to - just to do my daily things, like getting up and walking, going to the bathroom to get up and have my shower’.*

Recovery issues and priorities changed over time. In general, participants were most concerned with pain management and self-care during the acute recovery phase:

*P6 (Female, 70–74 years, fractured ribs).*

*‘My GP said it’s [the fracture] on your ribs, they’ll just heal between six to eight weeks. And that’s what happened. I did go on a lot of medication; it was very painful... But then with the time that went by I got better’.*

After the acute recovery phase had passed, participants’ priorities turned to resuming pre-injury daily life. Major barriers to further recovery at this time included chronic pain and persistent psychological symptoms.

### Perspectives and themes

**Fig. 1:**
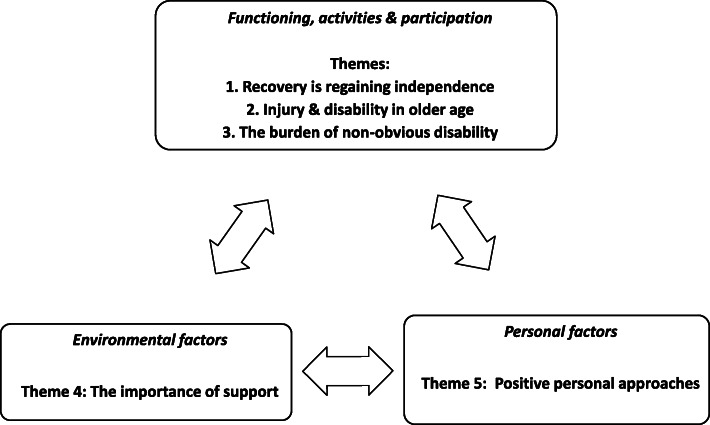
Content analysis themes for recovery from RTI in older age – an ICF-based approach

Five themes were identified in relation to the ICF conceptual model: recovery is regaining independence; injury and disability in older age; the burden of non-obvious disability; the importance of support and positive personal approaches (Fig. 1).

#### Theme 1: recovery is regaining independence

Regaining independence in pre-injury activities was a major facilitator of self-perceived recovery. Challenges to independence differed between individuals, types of injury and recovery phase. The initial acute recovery phase was characterised by dependence on others for self-care, such as eating, getting dressed and walking unaided. In the post-acute phase, returning to regular activities, including driving, were perceived as indicators of recovery. Whilst frustration was expressed regarding driving restrictions, being back in a vehicle also presented challenges. Illustrative quotes for this theme are presented in Table 2.

#### Theme 2: injury and disability in older age

Injury-related disability presented specific challenges in older age. Physical limitations and chronic pain had wide-ranging impacts on high-value activities such as caring for grandchildren, participation in weekly leisure and social activities and working life (which in some instances led to unplanned early retirement and financial concerns). The influence of older age on ongoing disability was raised. Illustrative quotes for this theme are presented in Table 3.

#### Theme 3: the burden of non-obvious disability

Late-onset physical disability, chronic pain and psychological injury were not readily apparent to others, but nonetheless had profound impacts on health and functioning (Table 4).

#### Theme 4: the importance of support

Practical and emotional support from family and friends was perceived as very helpful to recovery. Participants expressed a great deal of gratitude for the support they received from family, friends, community members and health care professionals. However, communication barriers with medical doctors were also mentioned. Participants who engaged with the compensation system had mixed experiences. Illustrative quotes for this theme are presented in Table 5.

#### Theme 5: positive personal approaches

Positive personal and / or psychological resources were important facilitators of recovery that also served as coping mechanisms in managing the experience of the injury itself and the recovery process (Table 6). The most prominent resources from the participants’ perspectives were determination: both to recover and to not let the injury stop them from living life; resilience; pragmatism; active coping strategies, e.g. adoption of physical and psychological adaptations and ‘work-arounds’ in order to regain functioning; being physically active; focusing on incidental positive outcomes (e.g. moving closer to family); selflessness; stoicism; realistic optimism; not taking oneself too seriously; a good sense of humour; being goal-directed; taking responsibility for one’s own recovery and health, and a positive attitude towards life in general. Illustrative quotes for this theme are presented in Table 6.

### Summary of key findings

A summary of the key findings from the study are presented in Table 7.

## Discussion

Our study explored older peoples’ perspectives, and factors related to recovery and health-related quality of life following a mild to moderate road traffic injury (RTI) in older age.

Five themes were identified: recovery is regaining independence; injury and disability in older age; the burden of non-obvious disability; the importance of support; and positive personal approaches. These themes coincidentally aligned with the key facilitators (regaining independence, support, positive personal approaches) and barriers (the presence of non-obvious disability, support barriers) to recovery and health-related quality of life identified in our analyses. This study meets a gap in our understanding of priorities and concerns for older people after mild to moderate RTI and revealed unique findings specific to older people. Recovery was defined as regaining independence post-injury and was viewed as more important than regaining pre-injury physical function. Positive personal approaches, including adaptation to changed health status, were both a facilitator of recovery and healthy coping mechanism for navigating both short- and long-term recovery and health.

Several of the findings from our study are consistent with those of studies that investigated RTI recovery in older people treated in the ED such as persistent pain, psychological injury, activity limitations, difficulties with activities of daily living, and changes to living situation [19–21]. Our findings also shared commonalities with RTI recovery studies in general adult populations, especially the importance of social support from family and friends; difficulties with loss of independence [39]; negative impacts on recovery due to chronic pain, anxiety and depression [24]; difficulties with independent self-care and /or domestic life, and reduced participation in recreational activities [24].

Two notable findings from our study have also been reported in the UK Impact of Injuries Study: difficulties with seeking and obtaining post-acute care and also with obtaining support from GPs for psychological symptoms [40]; and the extent of the major disruption the injury caused to all aspects of everyday life [41]. Our findings also shared some similarities with those of recovery from serious injuries, such as a positive and resilient attitude being a major facilitator of recovery [29]; an active (rather than a passive) approach to rehabilitation being beneficial to quality of life and functioning, [42] and negative impacts of injury on work and financial situation [43].

Our study revealed that whilst older people may share some similar recovery issues with younger people, each age group prioritises their recovery issues differently. For example, in younger people returning to work, recovering pre-injury physical health, mental health and financial stress are crucial to recovery. Our findings suggest that this is not the case for older people, whose primary concerns are around threats to independence. Older people also viewed positive personal approaches as integral to recovery.

Threats to independence identified by participants included not being able to drive, dress, eat or live independently, that is, any situation that resulted in a loss of autonomy in daily life. This finding is consistent with research into quality of life in older people by Ratcliffe et al., which found older people identify independence and control over daily life as particularly important to their quality of life [44]. An unexpected yet important finding of our study was the high value placed on regaining independence: it was considered far more important to recovery and health than regaining physical health. In fact, participants reported that incomplete physical recovery was acceptable if they could resume their pre-injury levels of functioning in daily life, even if adaptations or modifications were required, or the level of functioning was not quite as good as it was prior to injury. One possible explanation for the high value of independence is that older people view threats to independence as potential loss of capability and a barrier to further personal growth and well-being [45].

Participants demonstrated a wide variety of positive personal approaches that strongly facilitated self-reported health and recovery, such as resilience, pragmatism, a meaningful life where individuals contributed to society or helped others, a goal-oriented approach to recovery, being physically fit prior to injury, having a strong determination to recover and a high level of intrinsic motivation, especially with rehabilitation exercises. Furthermore, active coping strategies were mostly self-initiated without external guidance or support. This finding is consistent with evidence on resilience in older age, i.e. psychological resources such as having a higher sense of purpose in life; an optimistic outlook; a sense of control, and a flexible coping style able to persist with attainable goals, or redefine or replace unattainable goals [46].

In our study it was unclear whether resilience was associated with socioeconomic status. Evidence on resilience in the older population is also mixed: Windsor et al. reported social disadvantage as a key risk factor for resilience [46]; whereas Netuveli et al. found people aged 50 or more years who were resilient were more likely to have high social support than the non-resilient, but were otherwise not different socioeconomically [47].

Further insight into why the participants in our study appeared to cope well despite residual health issues is provided by a study by Mosser et al., which found older patients with heart failure reported better health-related quality of life compared to younger patients, that was not related to actual physical health, rather it was largely driven by changing expectations with advancing age as to what constitutes good health-related quality of life [48].

Our findings also share similarities with qualitative studies of recovery from hip fracture in older age. Pol et al. found that a positive attitude, strengths-based approach and emotional support was particularly beneficial for recovery from hip fracture, [49] whilst Young et al. investigated functional recovery one year after hip fracture and found self-determination played a significant role in making rehabilitation work [50]. Our findings are also consistent with evidence that resilience is a protective factor in people aging with a disability [51].

Establishing that our findings share many similarities with studies on injury recovery, disability and health-related quality of life in older age beyond RTI is an important finding, as it suggests opportunities exist to transfer well-established evidence from the wider literature to the specific issue of recovery from RTI in older age. This is especially the case for existing evidence on recovery from hip fracture, which could be applied to RTI in older age in the development of strategies and support to optimise health outcomes. Furthermore, utilising existing evidence (where appropriate) is more time and cost-effective than undertaking new studies.

Given independence is crucial to older people’s recovery and health-related quality of life following RTI, greater efforts are needed to help older people regain independence in physical and psychological functioning, driving, and / or living arrangements that allow for independent living without driving. Such efforts could include educating health professionals on the high priority older people place on independence compared to physical functioning; provision of appropriate services and identifying and minimising potential costs for those without insurance.

### Strengths and limitations

To the best of our knowledge this is the first qualitative study of recovery from mild to moderate RTI in older age. The participants were part of a larger, inception cohort study that was representative of all people living in NSW injured in a non-catastrophic RTI, and consisted of a variety of ages, road user types and injuries. This study was interested in recovery and health, from the time of injury to the present, so whilst for some studies recall bias is a limitation, this was not the case here. Although the sample size appears small (*n* = 12), interviews were continued until data saturation was reached, and evidence from a qualitative methodological study by Guest et al. demonstrated that 12 interviews are sufficient for studies that seek to understand perceptions and experiences,[52] as was the case with our study. Finally, the possibility that those who coped well post-injury would be more willing to be interviewed was a potential source of selection bias, especially given the influence of resilience on participants’ recovery journeys.

## Conclusions

Our study explored perspectives and factors related to recovery and health-related quality of life following RTI in older age and found a diverse range of types of injury, recovery experiences and long-term health outcomes. For some participants their injury was a minor event and temporary inconvenience, however for others it was life-changing and caused major disruptions to their lifestyle.

Five themes were identified: recovery is regaining independence; injury and disability in older age; the burden of non-obvious disability; the importance of support and positive personal approaches. Immediately after the injury, participants were most concerned with pain management and self-care, especially eating and dressing. Later, priorities turned to resuming pre-injury daily life.

Major barriers to recovery were threats to independence issues (especially driving and self-care), chronic pain and persistent psychological symptoms. Major facilitators of recovery were regaining independent functioning and positive personal approaches.

Older people’s recovery outcomes and health-related quality of life following RTI are improved by regaining independence. Greater efforts in helping older people to regain their independence are needed, and can be facilitated by health professionals’ attitudes, appropriate service provision and systems designed with this outcome in mind.

## Supplementary information

**Additional file 1.** Appendix 1 Sampling reference grid. A copy of the sampling reference grid used during the recruitment process

**Additional file 2.** Appendix 2 Interview guide. The guide used by the interviewer when interviewing participants.

## Data Availability

The data from this study is not publicly available and will not be shared as it contains potentially identifiable personal and sensitive information.

## Abbreviations

COREQ: The Consolidated Criteria for Reporting Qualitative Research (COREQ)
ED: Emergency Department
FISH: Factors Influencing Social and Health Outcomes following road transport injury inception cohort study
GP: General Practitioner
ICF: International Classification of Functioning, Disability and Health
NSW: New South Wales
mTBI: Mild traumatic brain injury
PTSD: Post-traumatic stress disorder
RTI: Road traffic injury
WHO: World Health Organisation
