# Supplementary material for: I’ve got to be independent’: views of older people on recovery following road traffic injury in New South Wales, Australia
Source: BMC Public Health. 2020 Aug 26;20:1294. doi: 10.1186/s12889-020-09391-0 (PMC7448973; doi:10.1186/s12889-020-09391-0)
Supplement: Supplementary file 1 — Additional file 1. Appendix 1 Sampling reference grid. A copy of the sampling reference grid used during the recruitment process [file 12889_2020_9391_MOESM1_ESM.docx]

## Appendix 1: Sampling grid

The following grid was used to ensure purposive sampling during recruitment.

**Purposive sampling goals**

- All people aged >=65 years at time of injury
- 7 people aged 65-74 years
- 7 people aged 75 years or above
- 7 male & 7 female
- At least 5 people living alone
- At least 5 people living with others
- At least 5 people with less than completed high school education
- At least 5 people who completed high school or above.

| Participant ID | Age (must be ≥ 65 years) | Sex (circle) | | Living arrangements (circle) | | Highest completed education level (circle) | | Consent (circle) | | Comment (optional) |
| --- | --- | --- | --- | --- | --- | --- | --- | --- | --- | --- |
|  |  | Male | Female | Alone | With others | High school or above | Less than high school | Yes | No |  |
|  |  | Male | Female | Alone | With others | High school or above | Less than high school | Yes | No |  |
|  |  | Male | Female | Alone | With others | High school or above | Less than high school | Yes | No |  |
|  |  | Male | Female | Alone | With others | High school or above | Less than high school | Yes | No |  |
|  |  | Male | Female | Alone | With others | High school or above | Less than high school | Yes | No |  |
|  |  | Male | Female | Alone | With others | High school or above | Less than high school | Yes | No |  |
|  |  | Male | Female | Alone | With others | High school or above | Less than high school | Yes | No |  |
