# Supplementary material for: I’ve got to be independent’: views of older people on recovery following road traffic injury in New South Wales, Australia
Source: BMC Public Health. 2020 Aug 26;20:1294. doi: 10.1186/s12889-020-09391-0 (PMC7448973; doi:10.1186/s12889-020-09391-0)
Supplement: Supplementary file 2 — Additional file 2. Appendix 2 Interview guide. The guide used by the interviewer when interviewing participants. [file 12889_2020_9391_MOESM2_ESM.docx]

## Appendix 2: Interview guide

**Warm up question**

Can you please tell me a little bit about the accident?

**General prompts**

**After the accident**

Did you go to hospital? If Yes:

- How did you get to the hospital (e.g. ambulance or car)? What was this like?
- How was the hospital stay for you?
- Were you able to move around in hospital?
- Can you please tell me about when you left hospital? What was that like?
- Did you go straight back home?
- Did you receive assistance or help from anyone?

If you didn’t go to hospital, can you please tell me about the treatment you received?

**Impact on others**

How did your family take the accident and your injury/injuries?

How are they now?

**Arriving home**

When you first arrived home, what was it like for you doing your usual daily tasks?

What impact did your health have on your usual daily life?

And how do you feel it impacted on those around you?

**In the present**

How are you going today, compared to before the accident?

And what about compared to just after the accident?

What about activities, such as hobbies or spending time with family and friends?

How do you now feel about driving or being a passenger in a car?

Has the accident changed how you get around every day, e.g. car vs public transport?

**Closing question**

For you personally, what is the biggest impact your injury / injuries have had on your day to day life?

That finishes our interview. Thank you very much for participating and sharing your experience.
